# Early Post-implementation Analysis of a Medical Student Mental Health Program

**DOI:** 10.1007/s40596-025-02193-9

**Published:** 2025-09-04

**Authors:** Robyn Bernstein, Kasey Cox, Claire Collins, Sujatha Changolkar, Kirk Brower, Erin McKean

**Affiliations:** 1 Naval Medical Center San Diego, San Diego, CA USA; 2 Western Michigan University School of Medicine, Kalamazoo, MI USA; 3 University of Kentucky School of Medicine, Lexington, KY USA; 4 University of Michigan, School of Public Health, Ann Arbor, MI USA; 5 University of Michigan Medical School, Ann Arbor, MI USA

**Keywords:** Medical student, Mental health, Counseling

## Abstract

**Objective:**

Mental health and burnout are major concerns among medical students, yet poor utilization of care persists. Barriers to care for medical students were identified in a previous study. Following this, a no-cost, confidential Medical Student Mental Health Program (MSMHP) was established to address common barriers to care. This study provides an analysis 1 year following the implementation of the MSMHP as well as a comparison to student attitudes and service utilization with the pre-implementation survey.

**Methods:**

In early 2023, a survey eliciting information regarding student burnout, barriers to care, and resource utilization and satisfaction was sent to 780 current medical students. Participation was anonymous and voluntary, with optional responses to each question.

**Results:**

Ultimately, 387 (50%) medical students responded. Burnout remained at similar levels between years (2020, 2.60; 2023, 2.59), as well as students reporting either a new or previously perceived mental health concern (2020, 67%; 2023, 65%). Satisfaction with the availability of mental health resources for medical students significantly increased since MSMHP implementation; 74% were “very” or “somewhat” satisfied in 2023, compared with 39% in 2020. While we found that the program addressed the barrier of access, barriers of stigma, time, fear of future disclosure, and cost remained.

**Conclusion:**

This study demonstrated that following the implementation of a MSMHP, a greater proportion of medical students obtained mental health care. Despite this, student burnout and concern over mental health and emotional well-being remained high. This may be explained by persistent systemic issues and barriers to care within the medical school experience.

It is well established that mental health concerns and burnout are major issues impacting the training of medical students [1, 2]. Many studies have been conducted looking at the levels of stress, burnout, and mental health conditions in medical students, especially during the COVID-19 pandemic, but few searched for tangible solutions. One study describes a “near-peer” support model in which senior medical students supported junior students during the pandemic through direct communication of mental and psychological issues and subsequent counseling on coping skills via stress management, exercise, contact with loved ones, and time management techniques. This model, which is utilized by many medical schools, appeared to provide a certain level of support [3]. However, these and other peer-support groups still leave a gap of care for students with serious mental health conditions requiring professional intervention, including access to mental health services and evidence-based individual counseling [4]. Additionally, most medical students nationwide do not seek treatment for their concerns; some studies show that fewer than 13% seek treatment [5].

A survey of medical students conducted at a US medical school sought to better understand barriers to seeking care, as well as desirable elements of a mental health program [6]. This study, conducted in 2020, showed that the largest drivers of accessing mental health care were lack of time, cost, stigma, fear of negative career impacts, and unawareness of resources. These results are consistent with other studies that have found stigma, and the potential for negative consequences, the cost of treatment, lack of time, and confidentiality concerns act as the main barriers to treatment utilization [7–9].

Following these findings, a partnership emerged between students and medical school leadership to create a dedicated Medical Student Mental Health Program (MSMHP). Over the course of 18 months, a workgroup consisting of students, deans, psychiatrists, and other stakeholders met regularly to develop a proposal and present it to executive leadership, which in turn authorized funding [10]. Accordingly, four full-time therapists were hired, along with extension of a full-time equivalent (FTE) psychiatrist, to provide mental health services exclusively for medical students. As far as documentation goes, students’ psychiatric records are maintained in the hospital-wide medical record system while therapy services are documented outside of the system-wide medical record. All services are free to enrolled medical students and include one-on-one counseling (limited to 12 sessions annually), therapy groups, seminars, and mental health screenings.

Within this context, we describe the follow-up study that was conducted 1 year after the new MSMHP implementation. We report levels of burnout, perceived mental health concerns, student utilization, barriers to treatment, and level of satisfaction with the new program. We also looked for improvement in service utilization between the two surveys.

## Methods

An online survey was disseminated to the medical student body in 2023, 1 year following the complete rollout of the program (i.e., both therapy and psychiatric services). Similar to the original survey conducted in 2020 [6], the 2023 cross-sectional survey was sent to a cohort of current medical students, including pre-clinical, core clinical, and clinical elective students; as well as those on a leave of absence and those participating in a dual-degree MD-PhD program. Each participant received an email inviting them to engage in the study voluntarily and anonymously. Respondents were able to skip any question. Participants were offered compensation with a $5 Amazon gift card. Although not all e-survey questions were identical in both years due to differing study objectives (needs assessment vs. program evaluation), some questions were consistent to assess change over time with differences described below.

Respondents were asked about concern for their emotional well-being and/or mental health during medical school (response options: yes; no; unsure) and whether they had ever sought treatment (response options: yes; no) or would seek treatment if they developed a mental health concern during medical school (response options: very likely, somewhat likely, neither likely or unlikely, somewhat unlikely, or very unlikely). If respondents endorsed that they would seek treatment or help, the survey asked where they would first turn to for help. Options included the MSMHP and other university, hospital, and community-based mental health service programs. If the respondents did not seek treatment for their perceived or diagnosed mental health concern, they were asked about barriers to treatment (response options: not enough time in my schedule; cost; didn’t know where to look for resources; tried but could not find an available provider; stigma; concern about documentation in the health system’s electronic medical record; worried they might be required to disclose such treatment during the course of their career; didn’t think it would work; or other).

All students were asked about their current level of satisfaction with the availability of mental health resources for medical students. Questions asking about the newly implemented MSMHP therapy services included whether the level of availability impacted their decision to attend this medical school and whether the availability of a similar program would factor into their decision for residency program selection. In order to gauge general student trends in pursuing mental health care, respondents were also asked to comment on historical utilization of mental health resources prior to this program’s implementation.

The survey utilized a non-proprietary single-item measure of burnout validated as a viable alternative to the Maslach Burnout Inventory (MBI) subscale on Emotional Exhaustion (MBI:EE) [11]. Each participant could choose one of the following: “1 = I have no symptoms of burnout, 2 = occasionally I am under stress but I do not feel burned out, 3 = I am definitely burning out and have one or more symptoms of burn out, 4 = the symptoms of burnout I am experiencing will not go away, 5 = I feel completely burned out.” Based on the 5-point scale, a response of ≤ 2 signified no symptoms of burnout, while ≥ 3 was scored positive for one or more symptoms of burnout.

The survey was administered using Qualtrics (Provo, UT). Analyses were conducted in R (Version 4.2.2; R Foundation for Statistical Computing). Means of the numeric burnout indicator were computed. Burnout levels are presented as both proportions and means. To assess whether burnout is independent of the curricular phase, Fisher exact testing was conducted. Because a table greater than 2 × 2 was assessed, Monte Carlo simulation was conducted. Bonferroni correction was applied to account for multiple comparisons. Chi-square testing was performed to test for significant differences in survey responses across the 2 years. Count and proportion analyses were performed to assess the prevalence of reported barriers such as time constraints, stigma, and cost.

## Results

Of the 780 recipients of the 2023 survey, we received 387 total responses (50% response rate). Table 1 illustrates the demographics of survey respondents. Medical students had a mean burnout score of 2.59 (SD 0.92), with the following categorization by clinical phase: pre-clinical students 2.32, core clinical students 2.74, and clinical elective students 2.66 (*p* ≤ 0.05). This was in comparison to the 2020 survey, where medical students had an average burnout score of 2.6, with the following categorization by clinical phase: pre-clinical students 2.75, core clinical students 2.72, clinical elective students 2.38 (*p* ≤ 0.01).

**Table 1:** Comparison of demographics of survey respondents: 2020 vs. 2023

Demographics^*^	2020 *n* (%)	2023 *n* (%)	*p*-value
Year in school			0.000
M1	70 (21.2)	93 (25.4)	
M2	93 (28.2)	80 (21.9)	
M3	110 (33.3)	75 (20.5)	
M4	28 (8.5)	73 (19.9)	
Leave of absence	11 (3.3)	25 (6.8)	
Medical scientist training program	18 (5.5)	19 (5.2)	
Gender			0.848
Male	107 (32.5)	114 (31.2)	
Female	218 (66.3)	245 (67.1)	
Not cis-gendered	4 (1.2)	6 (1.6)	
Race^҂^			0.000
African, African American, Middle Eastern	20 (5.6)	17 (4.4)	
Arab, Arab American, Middle Eastern	0 (0.0)	22 (5.7)	
Asian, Asian American, Desi	71 (20.1)	94 (24.3)	
European, European American, White	178 (50.3)	154 (39.8)	
Latinx	13 (3.7)	15 (3.9)	
Multiple	59 (16.7)	75 (19.4)	
Native American or Alaska Native	2 (0.6)	0 (0.0)	
Other identity, not listed above	11 (3.1)	3 (0.8)	

^*^Missing data has been excluded from the table; thus, category totals might not result in total survey respondents

^҂^Race response options differed between 2020 and 2023 surveys; direct comparison for all categories is not possible

Table 2 highlights survey outcomes of interest across the 2020 and 2023 surveys, including concerns about overall emotional well-being, concern about mental health, treatment utilization, barriers to treatment, and satisfaction with the availability of mental health resources. Across both surveys, most students (2020, 82%; 2023, 77.8%) reported having a concern about their overall emotional well-being at some time during medical school. Over half of participants (2020, 67%; 2023, 65%) reported either a new or previously perceived mental health concern. There was a statistically significant increase in the number of respondents seeking care in 2023.

**Table 2:** Comparison of major survey outcomes: 2020 vs. 2023

Survey outcome	2020 *n* (%)	2023 *n* (%)	*p*-value
Responded “yes” to: “Have you been concerned about your emotional well being at any point during your time in medical school?”	245 (81.9)	262 (77.8)	0.225
Responded “yes” to: “Have you been concerned about your mental health during medical school, either perceived or diagnosed by a professional? This includes both new and previously diagnosed mental health concerns.”	207 (67.4)	223 (65.4)	0.643
Responded “yes” to: “Did you seek treatment for this concern”	128 (61.8)	171 (76.7)	0.001
“Which of the following resources have you utilized”⇼			
MSMHP therapy* MSMHP psychiatry Peer support Personal therapist University-wide counseling services	N/A 66 (51.6) 8 (6.3) 68 (53.1) 24 (18.8)	98 (31.3) 83 (26.5) 12 (3.8) 89 (28.4) 31 (9.9)	N/A ⇞
“What prevented you from seeking treatment”⇼			0.229
Lack of time Concern about disclosure Cost Stigma Concern about documentation in EMR	44 (58.7) 27 (36.0) 25 (33.3) 23 (30.7) 19 (25.3)	44 (88.0) 13 (26.0) 14 (28.0) 14 (28.0) 9 (18.0)	
“What is your current level of satisfaction with the availability of mental health resources for medical students?” “Very” or “somewhat” satisfied	119 (39.0)	248 (73.8)	<0.001

^*^Not offered in 2020

⇞Unable to generate *p*-value across years due to differing resources offered

⇼Respondents were allowed to select more than one option for resources utilized and barriers to treatment, so reported percents will not sum to 100%

In 2020, 77 participants with self-reported mental health concerns identified barriers to obtaining care. With a similar response rate, this number decreased to 50 students in 2023. Sixty-nine percent of participants in 2020 and 88% in 2023 identified more than one barrier. Across the years, the most common reasons for not seeking care were not significantly different (see Table 2). Most respondents indicated a lack of time, stigma, fear of disclosure of treatment at some time during their career, and cost as barriers.

As shown in Table 2, satisfaction with the availability of mental health resources for medical students at the medical school increased from 2020 to 2023. Despite this, 93% of respondents in 2020 and 73% in 2023 thought the university should be doing more for their mental health. For respondents in 2023, the aspects of mental health services that students felt were the most important (“extremely” or “very” important) were the quality of services offered (96%); scheduling ease (93%); and having no negative impact on career (93%). When the response category was restricted to “extremely important,” the most highly endorsed item was the guarantee that seeking mental health care would have no negative impact on a student’s future career (76%).

A growing number of students endorsed the availability of mental health resources as impacting their decision to attend their current medical school based on the year of matriculation (3 respondents in 2020 matriculation, 12 in 2021, and 37 in 2022). The vast majority of students surveyed in 2023 felt that access to therapy services (90%) and psychiatric services (87%) similar to those offered through the MSMHP would factor into the ranking of residency programs.

## Discussion

This follow-up survey emphasizes the success of the MSMHP in terms of a greater proportion of students obtaining much needed care. Following a year after implementation of the MSMHP, the number of students not seeking treatment for their mental health concerns had decreased. Although the program has improved the percentage of people getting care for their mental health concerns, the percentage of students reporting a new or previously perceived mental health condition remained high, as did burnout. While burnout can reflect personal factors, underlying systemic issues can also drive mental health concerns, such as intense workload, high emotional burden in the line of work, and hierarchical power structures in medical training [12]. This may explain the respondents’ continued belief that the school should be doing more for mental health concerns, despite being generally happy with the resources provided.

Additionally, as an increasing number of students indicated that mental health resources were a significant factor in their medical school and/or residency decision, investing in mental health programs could potentially bolster recruitment of desired students and residents via increased applicant interest and application numbers. Aspects of mental health services that students felt important remained consistent between 2020 and 2023, including quality of services offered, scheduling ease, and no negative career impact. The data concerning negative career impact is consistent with the literature emphasizing that general stigma towards mental illness is a major concern of medical students in seeking mental health services [12, 13].

The results of our study should encourage the wider implementation of no- or low-charge mental health programs that supplement the classic peer-support model. The program requires a significant amount of buy-in and funding from school administration, which may be a barrier to implementation. However, not only does this benefit current and future students in access to quality, professional-level care, but it also addresses mandates laid out by the LCME in 12.3 and 12.4 to provide personal counseling to promote well-being and health care services, and encourages a culture shift to reduce the stigma of health professionals seeking treatment for their mental health concerns.

Study limitations include cross-sectional and overlapping student cohorts and vastly different mental health resources available during each time. A direct comparison between the two cohorts may also have been impacted by the COVID-19 pandemic. Even so, both groups engaged in the same curriculum and had similar levels of burnout. Moreover, this study does exemplify the importance of providing mental health resources for medical trainees. While it is difficult to generalize findings from a single medical school, each institution can be encouraged to conduct their own needs assessment, remedies, and follow-up studies.

Although implementation of the MSMHP has seen early success, and a potential model in the field of supporting medical student mental health, there are systemic factors driving mental health concerns in medical school and further improvements are needed. Programs must address issues such as limiting care to short-term therapy, and additional funding measures need to be acquired to either expand the number of appointments offered, available therapists, or access to outside services as a way to manage students’ continuing concerns about treatment costs. Other models for service delivery could also be added to the program, for example an opt-out, brief mental health screening for all first-year medical students as successfully implemented in other institutions [14].

Once the program has been in place for a more substantial amount of time, further investigations would be beneficial to better characterize common symptoms and improvements in specific quality of life measures related to the program. Specifically, it would be useful to ask medical students to identify therapy topics discussed during sessions (imposter syndrome, burnout, dealing with loss or grief, etc.) to gain a deeper understanding of patterns and how to best address them to optimize sessions. With continued support and expansion, the MSMHP seeks to continue to improve medical student mental health while in medical school.

## Data Availability

The data underlying this article cannot be shared publicly for the privacy of the individuals that participated in this study. The data may be shared for reasonable requests to the corresponding author.

## References

[1] Almutairi H, Alsubaiei A, Abduljawad S, Alshatti A, Fekih-Romdhane F, Husni M, et al. Prevalence of burnout in medical students: a systematic review and meta-analysis. Int J Soc Psychiatry. 2022;68(6):1157–70. 35775726 10.1177/00207640221106691

[2] Mirza AA, Baig M, Beyari GM, Halawani MA, Mirza AA. Depression and anxiety among medical students: a brief overview. Adv Med Educ Pract. 2021;12:393–8. 33911913 10.2147/AMEP.S302897 PMC8071692

[3] Rastegar Kazerooni A, Amini M, Tabari P, Moosavi M. Peer mentoring for medical students during the COVID-19 pandemic via a social media platform. Med Educ. 2020;54(8):762–3. 32353893 10.1111/medu.14206 PMC7267157

[4] Wilkes C, Lewis T, Brager N, Bulloch A, MacMaster F, Paget M, et al. Wellbeing and mental health amongst medical students in Canada. Int Rev Psychiatry. 2019;31(7–8):584–7. 31638441 10.1080/09540261.2019.1675927

[5] Puthran R, Zhang MWB, Tam WW, Ho RC. Prevalence of depression amongst medical students: a meta-analysis. Med Educ. 2016;50:456–68. 26995484 10.1111/medu.12962

[6] Collins C, Pichan C, McGee L, Siden JY, Brower K. Assessing student burnout, treatment acquisition, and barriers to care to prompt changes in a student mental healthcare program. Acad Psychiatry. 2023;47(2):164–8. 35879597 10.1007/s40596-022-01685-2 PMC9311347

[7] Knaak S, Mantler E, Szeto A. Mental illness-related stigma in healthcare: barriers to access and care and evidence-based solutions. Healthc Manage Forum. 2017;30(2):111–6. 28929889 10.1177/0840470416679413 PMC5347358

[8] Edwards JL, Crisp DA. Seeking help for psychological distress: barriers for mental health professionals. Aust J Psychol. 2017;69(3):218–25.

[9] Zaman N, Mujahid K, Ahmed F, et al. What are the barriers and facilitators to seeking help for mental health in NHS doctors: a systematic review and qualitative study. BMC Psychiatry. 2022;22:595. 10.1186/s12888-022-04202-9. 36071392 10.1186/s12888-022-04202-9 PMC9450826

[10] American Psychiatric Association: Center for Workplace Mental Health. Innovative Ideas: Medical Student Mental Health. Frontline Connect Toolkit: Mental Health for the Healthcare Workforce. 2023. https://vimeo.com/802822065/705c395365. Accessed 01 Feb 2025.

[11] Dolan ED, Mohr D, Lempa M, Joos S, Fihn SD, Nelson KM, et al. Using a single item to measure burnout in primary care staff: a psychometric evaluation. J Gen Intern Med. 2015;30:582–7. 25451989 10.1007/s11606-014-3112-6 PMC4395610

[12] Dyrbye LN, Thomas MR, Shanafelt TD. Medical student distress: causes, consequences, and proposed solutions. Mayo Clin Proc. 2005;80:1613–22. 16342655 10.4065/80.12.1613

[13] Hankir AK, Northall A, Zaman R. Stigma and mental health challenges in medical students. BMJ Case Rep. 2014;2014:bcr2014205226. 25183806 10.1136/bcr-2014-205226 PMC4158203

[14] Young C, Juliani M. Universal brief mental health screenings for first-year medical students: a 6-year retrospective of the Keck Checks program. Acad Med. 2023;98(7):782–7. 10.1097/ACM.0000000000005169. 36780668 10.1097/ACM.0000000000005169

